# Beyond practices and values: toward a physio-bioecological analysis of sleeping arrangements in early infancy

**DOI:** 10.3389/fpsyg.2015.00264

**Published:** 2015-03-12

**Authors:** Gianluca Esposito, Peipei Setoh, Marc H. Bornstein

**Affiliations:** ^1^Affiliative Behaviour and Physiology Lab, Department of Psychology and Cognitive Science, University of TrentoTrento, Italy; ^2^Division of Psychology, Nanyang Technological University, SingaporeSingapore; ^3^Eunice Kennedy Shriver National Institute of Child Health and Human Development, National Institutes of HealthBethesda, MD, USA

**Keywords:** sleeping arrangement, mother-infant interaction, cross-cultural research, co-sleeping, physio-bioecological

Should my baby sleep in my bed? There are clear reasons to do so, such as for warmth, comfort, bonding, and cultural tradition, but there are also clear reasons against doing so, such as increased risk of sudden infant death syndrome (Moon, 2011). Besides being a recurring practical question for parents, co-sleeping is a perennial academic issue as well. Hence, Shimizu et al.'s (2014) aim to examine “parenting practices and underlying cultural values of Japanese mothers” (p. 8) related to sleeping arrangements is timely and valuable. The authors predicted that mother-infant co-sleeping would decline from the 1960–1980s to 2008–2009 due to modern parents adhering to values that are more adaptive in an educated, urban, technologically and economically advanced society that has higher female participation in the workforce. However, they found equal prevalences for co-sleeping in Japan in the 1980s as compared to now. The authors suggested that this historical continuity is due to societal expectations which are in conflict with mothers' desire for gender egalitarianism. Here, we offer a complementary set of explanations as to why co-sleeping might be preserved among Japanese mothers over five decades of enormous social change, and we propose a direction where future studies on parent-child sleeping arrangements should go.

## The chronosystem: preservation of cultural practices

Research on parenting and social values usually focuses on which aspects of culture moderate parenting cognitions and practices and how they do so (Bornstein, 2012). In the face of rapid economic and sociodemographic developments around the world, developmental studies have begun to investigate how historical changes predict changes in parenting (e.g., Keller et al., 2005). Shimizu et al. based their hypothesis on the presumption that sociodemographic changes bring about changes in ethnotheories, and that ethnotheories exert a greater force on parenting than intergenerationally transmitted cultural values about the family and gender roles. Therefore, they expected that sociodemographic change would drive change in sleeping arrangements. However, Mindell et al. (2010) found co-sleeping for infants 0–36 months to be prevalent in predominantly Asian countries (64.7% as opposed to 11.8% in five predominantly non-Asian countries), and Japan was one of the 12 Asian countries surveyed. In Singapore, an economically advanced society like Japan, Mahendran et al. (2006) found that 33.1% of children aged 2–19 years (mean = 10.1 years) still slept with their parents. Hence, there is a lack of evidence in the literature that parenting practices have changed due to societal changes. It is more likely that sociodemographic changes do not necessarily bring about changes in parenting practices. Even granting the assumption that sociodemographic change predominates over conserved cultural values or local ecology, a still open question is: do socioeconomic pressures and transgenerational values affect change at the same rate? It is possible that socioeconomic forces do not change at the same rate as cultural values, but change faster. Thus, it is conceivable that the effects of socioeconomic and technological changes in Japan have not yet embedded themselves in everyday parental routines. Perhaps more families will use baby monitors that allow their infants to sleep in a different room, or robot nannies are in Japan's future (Chow, 2013).

## A multilevel approach to co-sleeping

Mother-infant interactions on a whole, including sleeping arrangements in early infancy, need to be examined and understood from a dynamic systems perspective, where individual-context relations guide the emergence and change in expectations, values, and practices at multiple levels of organization (Bornstein and Leventhal, 2015). Using a multilevel organization; the biological needs of the infant, physiological and psychological needs of the mother, and community/societal values and norms all must be considered to understand choices in parenting. By focusing on only one level—sociodemographic change, other levels that influence parents' decisions about infant sleeping arrangements may have been neglected. It is also unclear how each variable within the broad classification of social change influences parents' decision-making processes.

## A physio-bioecological proposal

Mammals share some similarity of brain mechanisms that constitute the neural bases of parental care. The greater development of cortical structures in humans make cognitions about parenting unique in humans compared to other mammalian species. However, we believe that to understand parent-infant dynamics, is it necessary to examine automatic physiological responses as well as complex cortical involvement during parent-infant interaction. Here we propose the need to approach parent-infant interaction in general, and sleeping arrangements in particular, following a physio-bioecological approach. Bronfenbrenner's (1995) bioecological theory depicts human development as embedded in an “ecological system” composed of five subsystems (microsystem, mesosystem, exosystem, macrosystem, and chronosystem). Each of these systems influences human development as well as knowledge, ideas, and values. A bioecological perspective considers the reciprocal interactions of human development and the multiple environments in which it unfolds (Cabrera et al., 2014). However, for the bioecological perspective to gain even greater purchase, we propose that it is necessary to couple it with a more in-depth assessment of the physiological mechanisms underlying specific behaviors (see e.g., Esposito et al., 2013). In the case of sleeping arrangements, a physio-bioecological approach should aim to answer three fundamental questions in the domain of early mother-infant interaction: (i) Physiological and biological characteristics: Parent and infant physiological responses to different sleeping arrangements. (ii) Macrosystem context: Caregiving practices and how these practices are influenced by cultural beliefs and parenting styles. (iii) Microsystem context: How different sleeping arrangements influence subsequent parent-child development (i.e., child's self-regulation, caregiver work-life balance, etc.).

Considering the need to examine sleeping arrangements from a multilevel perspective, we are inspired to take up Shimizu et al.'s call to more closely investigate sleeping arrangements and their implications for maternal-infant bonding and child well-being.

### Conflict of interest statement

The authors declare that the research was conducted in the absence of any commercial or financial relationships that could be construed as a potential conflict of interest.
